# Reducing risk of development or exacerbation of nutritional deficits by optimizing patient access to mealtime assistance

**DOI:** 10.1093/intqhc/mzz060

**Published:** 2019-12-22

**Authors:** SeÁn Paul Teeling, Heather Coetzee, Maeve Phillips, Mary McKiernan, ÉidÍn NÍ ShÉ, Aileen Igoe

**Affiliations:** 1 UCD School of Nursing, Midwifery and Health Systems, College of Health and Agricultural Sciences, Dublin 4, Ireland; 2 Mater Misericordiae University Hospital, Eccles Street, Dublin 7, Ireland; 3 Mater Lean Academy, Mater Misericordiae University Hospital, Eccles St., Dublin 7, Ireland

**Keywords:** Patient nutrition, Lean Six Sigma, feeding assistance, quality improvement, interdisciplinary

## Abstract

**Objective:**

Optimize patient access to mealtime assistance, decrease missed meal incidence, risk of malnutrition, reduce food waste and staff rework.

**Design:**

Lean Six Sigma methodology informed a pre/post intervention design.

**Setting:**

31 bed ward including Specialist Geriatric services and Acute Stroke Unit within an Irish University teaching hospital.

**Participants:**

Clinical and non-clinical staff including catering, nursing, speech and language therapy, dietetics and nutrition; patients, relatives.

**Interventions:**

An interdisciplinary team used the structured Define/Measure/Analyse/Improve/Control (DMAIC) framework to introduce visual aids and materials to improve the access of patients to assistance at mealtimes.

**Main outcome measures:**

Pre and post outcomes measures were taken for the number and cost of uneaten meals, rework for staff, staff and patient satisfaction, patient outcomes.

**Results:**

Following a 1-month pilot of a co-designed process for ensuring access to assistance at mealtimes, average wasted meals due to staff not being available to assist patients requiring mealtime assistance went from 3 per day to 0 corresponding to an average reduction of 0.43 kg per participating patient in food waste per day. Patients receiving assistance did not require additional oral therapeutic nutritional supplements, evidenced no new incidences of aspiration pneumonia or swallowing difficulties and were discharged without requirement for ongoing Dietetics and Nutrition support. Following a 6 month Control period comprising repeated PDCA cycles, the initiative was incrementally introduced to a further 10 wards/units, with positive feedback from patients and staff alike.

**Conclusion:**

The co-designed new process highlights the importance of staff and patient collaboration, inclusion and participation in designing quality improvement projects.

## Introduction

Malnutrition is an independent risk factor for complications, increased mortality, lengthened hospital admissions and healthcare costs [1]. The Irish Health Information and Quality Authority’s (HIQA) 2016 review of nutrition and hydration care in public acute hospitals identified one in four of admitted patients as being malnourished [2]. The location of this study, a major teaching hospital in Ireland, found that 25% of admitted patients identified as at risk of malnutrition fell into the high-risk category.

Feeding and swallowing are interconnected, as poor nutritional status is associated with the risk of swallowing difficulty [3] with corresponding risk of aspiration pneumonia [4]. While people on modified diets do not necessarily require feeding assistance, there are key patient groups who have swallowing difficulty, and are at high risk of having concomitant difficulties in self-feeding. Up to 80 % of people who have had a stroke have acute swallowing problems [5] and post stroke are at risk of hemiplegia and apraxia affecting ability to self-feed. Up to 47% of the frail elderly population over 70 years old admitted to an acute setting have dysphagia [6]. Merely increasing feeding support to all patients does not necessarily increase oral intake, but rather targeted assistance where required is recommended [3].

The economic consequence associated with malnourished patients in Ireland in 2007 was estimated to be in excess of €1.4 billion, representing more than 10% of the healthcare budget that year [7, 8]. Additionally, patient expectations of hospitals continue to rise thus increasing the focus on food provision in hospitals [2]. There is little in the literature about the practicalities of food provision in hospitals or the challenges for ward staff in offering assistance [2, 9, 10]. However, there is recognition that feeding assistance is time consuming with barriers being identified including insufficient time in the wards and insufficient staff [10].

In November 2011, a food waste survey in the study site [11] conducted by Clean Technology Centre Cork (CTC) measured volumes of food waste quantities for each meal from:

- a hospital wing (12 wards)
- the staff restaurant
- the main kitchen

Total hospital food waste was estimated at 119 tonnes annually, with 43 tonnes of food waste attributed to the hospital wing surveyed, and a further estimate of 31 tonnes for wards not surveyed. Significantly, 62% of food waste was at ward level with associated costs of €129,000 per annum. The actual food wastage for the pilot ward of this project was measured as 13.2 kg daily (breakfast – 4.02 kg, lunch 4.67 kg and tea 4.55 kg) equating to an average of 0.42kg-wasted food per bed. Given the knowledge of the impact of missing meals on patient outcomes and costs [2, 7, 8] a key objective for the hospital was to identify the root cause for food not being eaten. As a response a team was convened comprising representation from nursing, clinical dietetics and nutrition, speech and language therapy (SLT), and a catering manager as the team lead. The team focus was to optimize patient access to good nutrition and reduce corresponding ward based food waste.

## Methods

### Choice of a Lean Six Sigma approach

‘Lean’ describes the philosophy underlying the Toyota Production System (TPS) [12–14] that was developed in the Japanese car manufacturing industry. It is a quality improvement approach that consists of the elimination of waste to improve the flow of people, information or goods—in a hospital setting this would improve the flow of the patients through the system [15–17] by eliminating Non Value Add (NVA). This activity can include anything from waiting for an appointment, the actual procedure or diagnostic, or for any interaction with a member of the hospital team, both clinical and non-clinical. Six Sigma is a concept that was originated by Motorola Inc. in the 1980s and is a data driven process improvement methodology designed to improve process capability and enhance service delivery by the introduction of improvement projects focusing on eliminating process variation [18, 19].

Synergies were seen to exist between both Lean and Six Sigma as they each take a process view and converge in their focus on variation, flow and the customer [20]. A combined Lean Six Sigma (LSS) approach looks for ‘root causes’ of problems with real time observational data collection carried out in the workplace, [22] referred to as ‘Gemba’ in LSS terminology. Within the literature LSS projects in healthcare have outlined the applicability of LSS principles due to its zero tolerance for mistakes and potential for reducing medical errors [22, 23], however, there is a gap in the literature of the impact of Lean [18, 19, 23] which continues to be analysed for its overall impact on staff and patient wellbeing.

In 2011 the study site commenced its initial phase of staff training in LSS principles to look at ways of improving patient and staff experience and patient outcomes. LSS was chosen as the methodology of choice as the CEO of the time had experience of its successful application in a healthcare setting. Hospital based teams used LSS methodology as part of their progress towards Lean Six Sigma qualification.

### Use of LSS methodology in this project

Six Sigma gives structure to process improvement through a series of defined steps [19, 20, 24] known as DMAIC:

- Define the problem
- Measure the problem—gather data that illustrate the problem
- Analysis of the problem data to discover root causes
- Improvement of the problem through data driven solutions
- Control to prevent reoccurrence through monitoring of the data

In the Define phase the team developed a detailed project Charter, a robust one page document stating the problem, scope, goal, timeline, team, business case and to identify the ‘customers’ of the Project. Using LSS tools, the scope was narrowed to a pilot ward, in line with the CTC waste survey and to facilitate a sample size for patient, relative and staff engagement that met SMART (Specific, Measureable, Achievable, Relevant, Time-bound) objectives for project delivery. The ward chosen was a 31 bed medical ward that included part of the Specialist Geriatric Ward and the Acute Stroke Unit and one of the highest for daily food waste (13.2 kg per day), with a demographic of patients with risk factors for malnutrition corresponding with those in the literature [4–6].

To facilitate the cohesiveness of the team, consolidate the Project Charter and to make the project objectives clear, use was made of a simple LSS tool called a SIPOC (Suppliers, Inputs, Process, Inputs, and Customers) [25]. The SIPOC allowed a high level overview of the process of patients receiving their meals. The tool also facilitated the project team discussion on stakeholder engagement required to initiate and sustain any required change of practice at mealtimes, to enable a process of patients receiving the appropriate meal with required assistance in a timely manner.

The process of the journey of a patient meal following diet recommendation, from start (identifying those requiring assistance) to finish (patients having their meal) was initially process mapped following qualitative interviews with the relevant stakeholders to determine how they thought the process currently worked ‘what we think it is’. It was noted as important that the hospital operated a ‘cook chill’ system, defined as:

‘A catering process whereby meals or meal components are fully cooked, then cooled by controlled chilling, e.g. blast chilling, and subsequently stored at a temperature above freezing point (3°C) prior to regeneration and/or service.’ [26]

This system of food provision means that the correct meal order is essential and taking time to go through dietary requirements with the patient and understanding their ability to eat is paramount.

**Figure 1:**
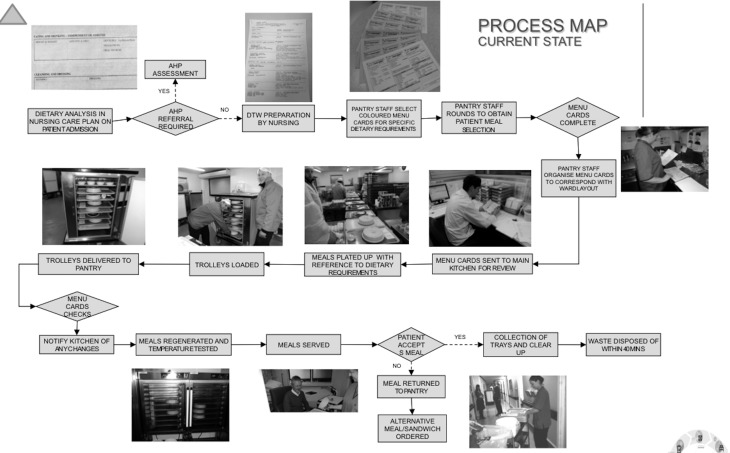
‘As is’ Process Map (current state).

Following this, the Lean team undertook a number of observational studies (Gemba [21]) to gather real time observational data. This data facilitated the refining of the initial process map of the patient meal from the previously identified start and finish points and identified any missed or additional steps not identified in the ‘what we think it is’ stakeholder engagement stage to develop an ‘as is’ or current state map (Fig. 1).

With this map the LSS Lean team were then able to locate areas of potential NVA and identified the following data for collection over a scheduled 2 week period (Monday-Friday):

1. Number of uneaten meals: 42% of patients (*n* = 13) on the 31 bed pilot ward did not eat a hot meal at the main mealtime. Further analysis of the number of uneaten meals revealed the following root causes:

- 23% of patients were actually too ill to eat.
- 24% of patients required assistance that was not immediately available
- 15% of patients were off the ward at the mealtime
- 38% of patients were fasting for a diagnostic or interventional procedure

The remainder of patients on the ward (58%) could eat their meals independently and were excluded from the intervention.

2. Cost of uneaten meals: A single meal including labour costs equates to €4.57. Quantifying the cost of meals wasted for the patient cohort requiring assistance (24%) and extrapolating this figure across all 26 wards/units (based on staffing and patient demographic) the estimated cost per annum of wasted meals for this single cohort was €18 535.92.

3. Cost of expertise: Key interactions in relation to determining optimum patient nutrition took place with Nursing, SLT and Clinical Dietetics and Nutrition staff. Working from the mid-point of the scale it was estimated that associated cost of healthcare professional input when a meal is wasted was €79.18 per patient covering duplication of nursing staff time, and Speech and Language Therapy and Dietetics and Nutrition specialist review.

4. Rework: TIMWOODS [27] is a framework used to identify NVA or process waste under the headings—Transport, Inventory, Motion, Waiting, Over processing, Over production and Skills. Applying TIMWOODs to the Gemba observations, the following was noted:

- Transport—missed meal reorder (mean transit time of 15 minutes)
- Inventory—meals cannot be reheated [26]. A second meal is ordered incurring cost.
- Motion—additional motion in disposal and re-plating
- Over processing—additional phone calls between departments
- Over production—allowance for additional meal requirements
- Defects—incorrect meal or incorrect form of meal
- Skill—wasted time and skill of staff

*Significantly, it was noted by the catering team that due to the cook chill system a replacement meal might not always be immediately available.*

The ‘as is’ process map and the data collected on the pilot ward were then shared with the stakeholders to gain their perspective.

Following further SMART review, Stakeholders and the LSS Lean team agreed that the scope of the Project should be narrowed to focus specifically on the cohort of patients who required assistance that was not immediately available (24% of those who missed a meal). The cohort of patients who did not eat a meal due to diagnostics, interventions, fasting and actually being too ill to eat were considered areas outside of scope as they were identified as either common cause (diagnostic, intervention, fasting) or special cause (too ill to eat) variation. In light of this and following use of several LSS brainstorming tools including Affinity mapping [22]—the following issues with assisted feeding were identified:

#### Ward level

- No consistent way to identify which patients required assistance
- Ad hoc Nursing and Health Care Assistant (HCA) breaks as reacting to demand for assistance
- Staffing numbers meant less time to spend assisting the patient

#### Catering department level

- Rework

#### Dietetics and speech and language therapy level

- Impact on patient when meals not served
- Increased risk of swallowing issues when assistance is not available
- Increased risk of malnutrition if meals missed

**Figure 2:**
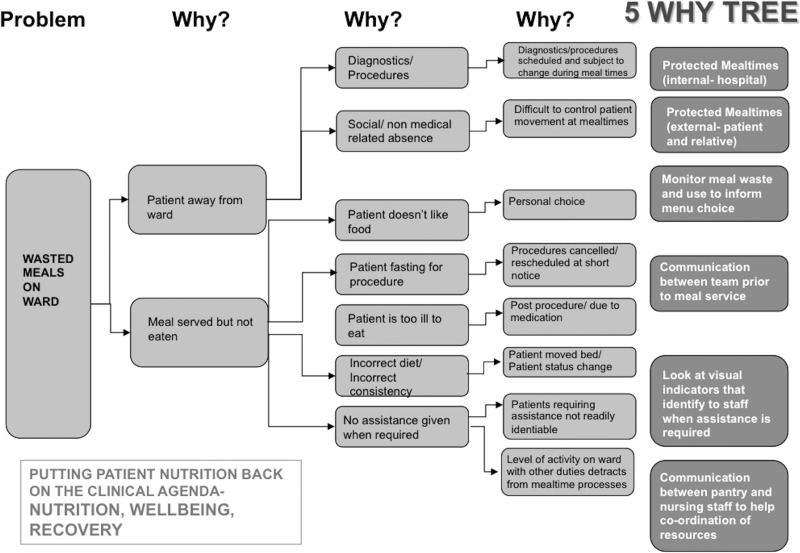
5WHY tree to facilitate root cause analysis.

The group then utilized a 5 Why root cause analysis tool (Fig. 2) to ascertain root causes and solutions for the identified problems.

At 4 Whys, high level potential solutions identified with stakeholders included:

- Improve communication between health professionals and catering Staff
- Potential visual indicators to assist in managing patients who required assistance
- Meal waste monitoring

**Figure 3:**
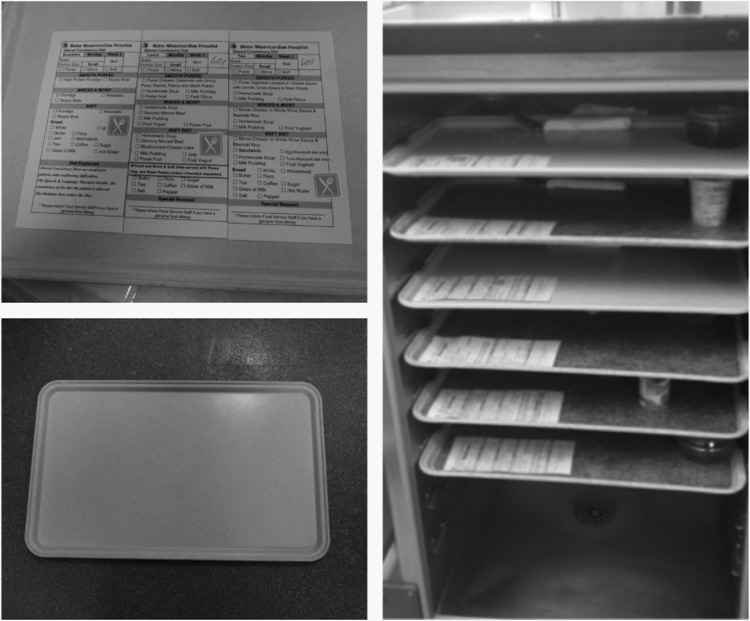
Visual aids.

A further stakeholder session took place and the following process changes were agreed:

- The existing dietary census at ward level would now have a simple ‘A’ (signifying assistance) handwritten next to patients which the Nursing team identified as needing assistance based on the baseline clinical assessments. This did not add significantly to nursing workload as it took less than a minute. As it was handwritten it also did not require the dietary census to be remodelled.
- All nursing, pantry and catering staff would be made aware of the significance of the ‘A’
- Ward pantry staff would place a new green sticker (Fig. 3) on a patient dietary request card (regardless of type of diet) if the patient had an ‘A’ next to their name on the dietary census which would indicate to catering staff in the main kitchen to plate these on a green tray
- The catering department would plate all meals for patients with a green stickered dietary card on a corresponding green tray (Fig. 3) that contrasts with the standard grey tray. One of the Lean team had visited the Pembury hospital in Kent where he had witnessed coloured trays in use at mealtimes and this seemed like a transferrable visual indicator that could be useful for use as part of any solution.

The above solutions enabled Nursing staff to immediately identify by visual indicator of tray colour the number of patients requiring assistance with their meals and stagger the staff breaks accordingly. The catering department agreed to stagger the regeneration of the cook chill meals to facilitate the availability of Nursing and HCA staff to assist the patient.

**Figure 4:**
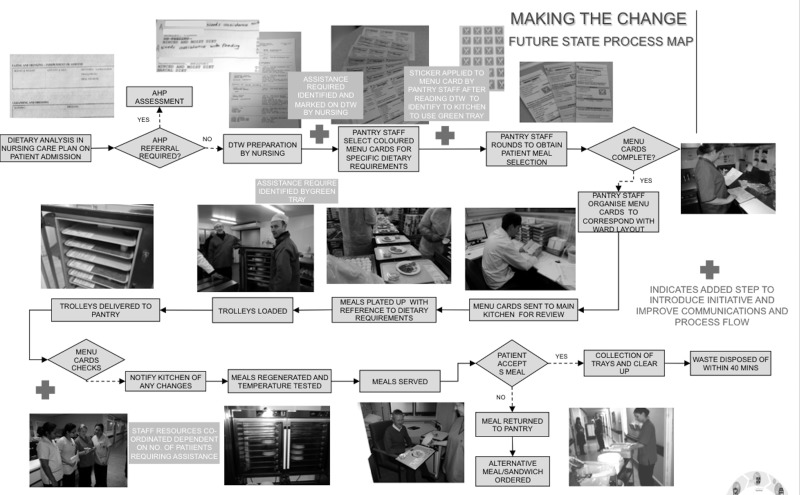
Future state process map.

The agreed new process was integrated into a ‘future state’ process map (Fig. 4), which was agreed and signed off by the Lean team and relevant stakeholders for the pilot ward.

Extensive training took place on the new process with all the relevant staff that interacted in any way with meal provision on the pilot ward. This took place in the form of lunchtime/break sessions with use of aids such as the sticker, green tray (Fig. 4) and a walk through the process map. The Lean team also carried out an additional training session for 60 catering staff on other wards/units so that the process would be familiar to all of the team before it started.

## Results

### Study outcomes

The Pilot was commenced for 1 month and monitored by the Lean team on a daily basis. During this period (allowing for ongoing patient admission and discharge to the ward) an average of 7 patients received 21 meals (breakfast, lunch, tea) on a green tray on a daily basis. Within 1 month of the implementation of the Project on the pilot ward, the average wasted meals wasted due to staff not being available to assist these patients at mealtimes went from 3 per day to 0. This also equated to a reduction of 0.43 kg per patient receiving required assistance, in food waste per day. Due to the ability to immediately visualize the requirement for assistance, the Nursing team could further anticipate requirements ensuring no patient was waiting who required assistance.

Patients who received their meals on a green tray showed improvement in their nutritional status, evidenced by:

- Receiving assistance to eat the appropriate meal as advised by dietetics and nutrition resulted in no requirement for these patients to access additional oral therapeutic nutritional supplements
- No patients requiring assistance had any incidence of an adverse Malnutrition Universal Screening Tool (MUST) score where indicated
- All patients in this cohort were discharged without requirement for ongoing Dietetics and Nutrition support. In addition, there were no new incidences of aspiration pneumonia or other sequalae of swallowing difficulties reported.

#### Feedback on results

Feedback was sought from immediate stakeholders via a follow up focus group [n = 18] that indicated success factors as below:

#### Staff

- No new paperwork but using existing dietary census form
- Simplicity of green sticker
- Easy identifiable green tray
- Baseline data to support case
- Good teamwork

#### Patients and relatives

Patients and their relatives who had received a meal on a green tray were asked to participate in an on ward qualitative interview over a further 1-month period. 100% of patients (*n* = 33) and relatives (*n* = 28) surveyed were happy that there was always a nurse or HCA to assist them, with 100% of relatives saying that they felt confidence in the green tray system.

#### Cost

The campus development team sponsored the purchase of green trays (n = 200) that have remained in circulation since, effectively making their supply cost neutral. Stickers were incorporated into the existing catering department budget (10 cent per unit) with cost savings from reduction in wasted meals (1 meal including labour cost at €4.57) covering these. No additional staffing resource has been required to deliver this improvement.

In order to embed the Project it was maintained on the Pilot ward for a period of 6 months, with monthly PDCA (Plan, Do, Check, Act) cycles carried out by the Lean team to monitor the control phase. This allowed time for the new process to become ‘business as usual’.

## Discussion

Currently the project has rolled out to all applicable wards and units in the hospital. Some high acuity/dependency units where patients have a 1:1 nursing ratio do not need signalling for additional assistance, so the trays are not required in these areas. The trays are deployed to maximum affect in ward/units that do not have these staffing ratios. The meal wastage is audited daily by the Pantry staff and weekly by the catering supervisors, so any spike in food wastage immediately alerts the ward that a tray may have been required. The noted reduction in the number of missed meals by patients who required assistance, reduced need for therapeutic nutritional supplements and no additional incidences of aspiration, were seen as key results of this project with the additional and welcome effect of cost savings. The expertise of the initial assessment of these patients by Speech and Language Therapy, Dietetics and Nutrition and Nursing staff is clearly not wasted as the prescribed dietary requirements and composition are received by the patients as prescribed.

**Figure 5:**
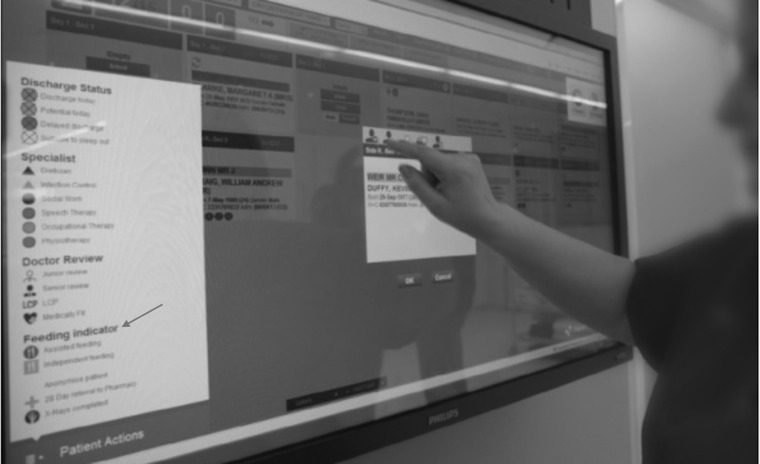
Patient flow board.

A limitation of the study was noted as focusing on only 24% of patients - those who required assistance. Potential bias to other patients such as those who were off the ward for diagnostics (38%) or were not on the ward at mealtimes (15%) was seen as potentially being removed if these patients could benefit from a ‘held’ meal (not regenerated [26] until needed) if staff knew about it in advance. Building on this feedback, in 2014 when the hospital introduced a real time patient flow system, giving real time status as to patients and noting planned diagnostic procedures, the input of the project team into this development enabled a notification system for patients who are off the ward and may need meals held (although not requiring assistance and if not contra indicated e.g. if the patient were fasting). Subsequently the system has incorporated the green sticker symbol onto the flow system touch screen to enable staff to identify patients requiring assistance at a glance (Fig. 5).

The key strength of the project was the inter and multidisciplinary nature of the team yielding a robust effective team, which quickly overcame any silo thinking and engaged and supported each other with a high challenge/high support approach. The clinical nurse Managers on the pilot ward and nursing management wholeheartedly supported the project from its inception. This brought other stakeholders at ward level on board. The catering manager who was the project lead, lead from the front with enthusiasm and expertise. This enabled the project having an identified ‘owner’ in that the catering department agreed to continue running and monitoring the ‘green tray’ if the pilot proved successful, which would contribute to roll out to other units and to project sustainability. It should be noted that new ways of working and new processes take time to adapt. The education sessions and focus groups organized and delivered by the Lean team certainly helped the success of the pilot. However, it was noted that this did require additional time and commitment from the team who were already training in LSS in addition to their substantive posts.

## Conclusions

The improvement has been successful in identifying and consistently providing assistance for patients who require support at mealtimes. For patients this effectively eliminated the risk of development or exacerbation of nutritional deficits resultant from lack of access to feeding assistance and missed meals. There is more efficient use of staff time and skill by reducing duplication consequent to re-ordering meals and increased review or intervention when patients do not receive meals. The project is consistent with the study sites approach to ‘food first’ with the emphasis on meals before supplements, as echoed in the recent national food, nutrition and hydration policy [28]. There has also been reduction in food waste. When it comes to change, simple practical schemes are more likely to be successful than complex ones. A significant contribution to the success of the project is that there was a multidisciplinary team involved from the beginning that actively supported the scheme.

This project illustrates that the implementation is just one element of a successful project. The access to LSS methodologies for the team resulted in clear scope of project, structured exploration of root causes for the issue identified, and a clear implementation plan for quality improvement. LSS thinking supported successful leadership on change, engaging and supporting each other and the relevant stakeholders to ensure project sustainability.
